# The Scaling of Human Contacts and Epidemic Processes in Metapopulation Networks

**DOI:** 10.1038/srep15111

**Published:** 2015-10-19

**Authors:** Michele Tizzoni, Kaiyuan Sun, Diego Benusiglio, Márton Karsai, Nicola Perra

**Affiliations:** 1 Computational Epidemiology Laboratory, ISI Foundation, via Alassio 11/C, 10126, Torino, Italy; 2 Laboratory for the Modeling of Biological and Socio-technical Systems, Northeastern University, Boston MA 02115 USA; 3 Dipartimento di Fisica, Universitá degli Studi di Torino, via Giuria 1, 10126, Torino, Italy; 4 Laboratoire de l’Informatique du Parallélisme, INRIA-UMR 5668, IXXI, ENS de Lyon, 69364 Lyon, France

## Abstract

We study the dynamics of reaction-diffusion processes on heterogeneous metapopulation networks where interaction rates scale with subpopulation sizes. We first present new empirical evidence, based on the analysis of the interactions of 13 million users on Twitter, that supports the scaling of human interactions with population size with an exponent γ ranging between 1.11 and 1.21, as observed in recent studies based on mobile phone data. We then integrate such observations into a reaction- diffusion metapopulation framework. *We* provide an explicit analytical expression for the global invasion threshold which sets a critical value of the diffusion rate below which a contagion process is not able to spread to a macroscopic fraction of the system. In particular, we consider the Susceptible-Infectious-Recovered epidemic model. Interestingly, the scaling of human contacts is found to facilitate the spreading dynamics. This behavior is enhanced by increasing heterogeneities in the mobility flows coupling the subpopulations. Our results show that the scaling properties of human interactions can significantly affect dynamical processes mediated by human contacts such as the spread of diseases, ideas and behaviors.

The network of social interactions between individuals in a community represents the main substrate for a number of spreading phenomena such as the diffusion of infectious diseases, ideas and behaviors^1^,^2^,^3^. In the past fifteen years network science has developed a wide range of mathematical tools to study and model such dynamical processes^3^,^4^,^5^. In particular, building upon a long research tradition in ecology^6^, the theoretical framework of reaction-diffusion (RD) processes on metapopulation networks has been proved to be extremely valuable for describing contagion phenomena in spatially structured systems^7^. In this framework, individuals are represented by particles that reside in nodes of a network and migrate along the connections between them. Each node describes a subpopulation, i.e. a city or a town, while each link represents a travel route. Inside each node, particles react according to the rules of the process under study. Such modeling approach has been widely used to describe the dynamics of a number of real world complex systems^8^,^9^,^10^. Its most successful application, though, has been the modeling of the spread of infectious diseases in structured populations^11^,^12^,^13^,^14^,^15^,^16^,^17^,^18^,^19^,^20^,^21^,^22^,^23^,^24^. A common assumption in RD metapopulation models is that particles interact in each node with the same contact rate, constant and equal for any given size of the subpopulation. In mathematical epidemiology, such assumption corresponds to the frequency-dependent transmission rate^25^. However, in the past years, considerable efforts have been devoted to quantitatively measure human mixing patterns in a variety of settings, from small spatial and temporal scales^26^ to country wide studies^27^. This has been possible thanks to the availability of new emerging technologies^28^, such as RFID sensors^26^, mobile phones^29^ and social media^30^. A recent study based on the analysis of large mobile phone datasets^31^ has shown evidence that the *per capita* social connectivity scales with the subpopulation size. In particular, the authors of^31^ found that the cumulative number of social contacts of individuals in a city scales as 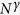 where 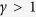 and *N* is the city’s population. This finding is consistent with a number of scaling properties observed in cities such as wages, crime rates, infrastructure per capita^32^,^33^ and with theoretical models of urban development^34^,^35^.

In this work, we first present new empirical evidence, based on the analysis of human interactions on Twitter that supports the contacts scaling hypothesis. Then, we integrate such observation into a RD metapopulation framework characterized by realistic heterogeneities in the distribution of the number of connections per node and in traffic flows. In particular, we study a Susceptible-Infectious-Recovered (SIR) epidemic dynamics inside each subpopulation^36^. We provide an explicit analytical expression for the global invasion threshold that sets a critical value of the diffusion/mobility rate below which a contagion process is not able to spread to a macroscopic fraction of the system^17^. We show that the scaling of interaction rates with subpopulation size significantly alters the contagion dynamics leading to a lower critical value of the mobility rate. Interestingly, such variations are enhanced by increasing heterogeneities in mobility patterns coupling the subpopulations.

## Results

### The scaling of human contacts on Twitter

We analyze the interactions between users of the micro-blogging platform Twitter in several countries. We considered two different geographical aggregations (see Material and Methods for more details). The first maps about 13 millions Twitter users into 2,371 census areas centered around major transportation hubs^12^ in 205 countries. Such aggregation level has been used to model pandemic spread at the global scale^13^,^24^. The second maps about 4.6 million Twitter users into 1,344 metropolitan areas, across the USA and 31 European countries.

To extract the relation between contacts and population size, we follow the methods used by Schläpfer *et al.*^31^. In both aggregation levels we build the communication network through Twitter mention interactions (see Material and Methods). In our analysis, a link is placed between users *A* and *B* within a given census area if and only if *A* mentioned *B* and vice versa at least once. We calculate the *cumulative degree* 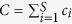, *where c*_*i*_ *is the degree of user i and S is the number of users within a census area, and rescale it by the users’ coverage* 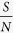 *to obtain* 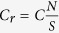
*, where N is the total population of a census area obtained from official sources*^37^,^38^,^39^. *The rescaling procedure corresponds to an extrapolation of the observed average nodal degree,* 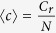
*, to the entire population of the census area^31^, and effectively reduces fluctuations due to variations in coverage from city to city.*

**Figure 1:**
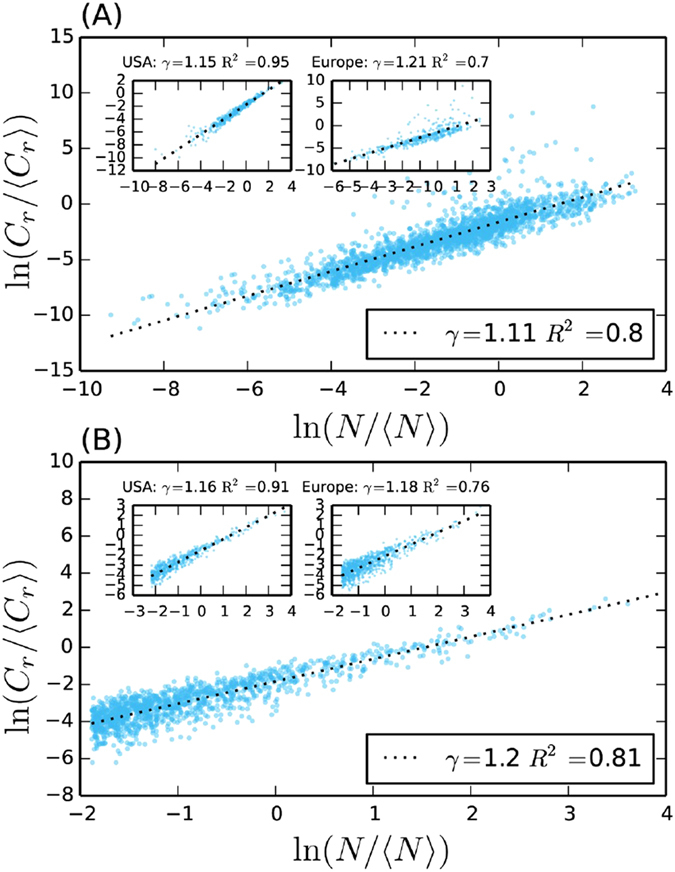
(**A**) Rescaled cumulative degree *C*_*r*_ against population *N*, measured between 13129406 Twitter users distributed across 2371 basins in 205 countries. (**B**) Rescaled cumulative degree against population, measured between 4606444 Twitter users in 1344 metropolitan areas in 31 countries. We normalized the values of *C*_*r*_ and *N* by their average to compare the results across different countries. Insets show the dependency of *C*_*r*_ on *N* restricted to the Twitter users in the US and Europe.

*To test the scaling hypothesis, we fit the rescaled cumulative degree C*_*r*_ *to a power-law function of the population of the census area, in the form* 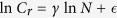, *and compare the result against a null model, represented by a linear function of the population,* 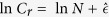, *where* 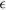 *and* 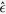 *are constant. In all cases the power-law function is found to be a better fit to the data than the linear regression, based on the adjusted R*^*2*^*, and the difference between the exponent* 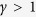 *and the simple linear regression is statistically significant* (*p* < 1.001*, details in the Supplementary Information file*). More specifically, we find, consistently with Schläpfer^31^, that the rescaled cumulative degree *C*_*r*_ is characterized by a power-law relation with the population of the census areas, 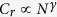 with exponent 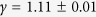 considering basins and 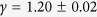 considering metropolitan areas (see Fig. 1). We also restrict our analysis of the Twitter dataset to the two aggregation levels in the USA and Europe. We find that the scaling behavior still holds, with the exponent *γ* in the same range, i.e. 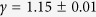 in the USA and 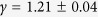 in Europe considering census areas, and 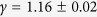 in the USA and 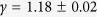 in Europe considering metropolitan areas.

Similar results are obtained considering also the connections of a user in the whole network. In particular, when the total number of Twitter interactions *C* is calculated by assuming *c*_*i*_ to be the degree of user *i* in the entire network, *c*_*i*_ is no longer confined within the basin/metropolitan area boundary and the interactions between user *i* and users from other basin/metropolitan areas or users that are not geo-mappable are also taken into account (see Fig. S2 in the Supplementary Information file). In this case, we find 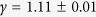 in the US, 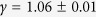 in Europe and 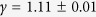 when considering all the basins in the world. For the case of metropolitan areas, we find 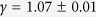 in the US, 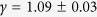 in Europe and 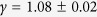 when combining all the metropolitan areas of the US and Europe together. In Table 1 we report a complete summary of the values of *γ* computed at all scales and aggregation levels.

### Global invasion threshold and numerical simulations

To study the effect of the scaling of contact rates in RD processes, we consider a metapopulation network of *V* nodes, and *N* individuals. Each node *i* has degree *k*_*i*_, and population size *N*_*i*_(*t*). The degree describes the number of subpopulations connected to it. We adopt a degree-block approximation, assuming all the subpopulations of degree *k* to be statistically equivalent^15^,^16^,^17^. We denote the degree distribution of the network as *P*(*k*). To describe the diffusion of individuals, we assume that the rate at which individuals leave a subpopulation is independent of its degree and equal to *p*. However, to reproduce the properties of real transportation networks^40^, we consider heterogeneous distributions of degree and traffic flow. In particular, the diffusion rate of individuals between two nodes of degree *k* and 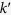 is 
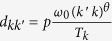
, where *T*_*k*_ provides the necessary normalization, and *ω*_0_ is a system dependent constant that rescales the diffusion rates between nodes. Without lack of generality we set *ω*_0_ = 1. It is possible to show that, under such conditions, the population of a node of degree *k*, 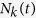, at equilibrium is given by 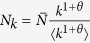, where 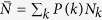
17. As a consequence, the exponent *θ*, which modulates the heterogeneity of the mobility flows, also regulates the heterogeneity of the subpopulations size distribution. We model the reactions, taking place in each node, as a stochastic SIR epidemic process where individuals are partitioned according to their health status: susceptibles (S), infectious (I) and recovered (R). The SIR dynamics are defined by two transitions: the infection process 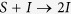, regulated by the transmissibility λ, and the recovery process 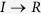, tuned by the recovery rate *μ*^2^.

Here, we investigate the case in which the infection dynamics is dependent on the local population size. More precisely, inside each node, we consider an homogeneous mixing approximation where the average contact rate scales with the population size as 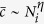. The values of the exponent *γ* measured in real social networks correspond to 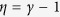 ranging between 0.11 and 0.2. The value of is η measured by Schläpfer *et al.*^31^ *is η* = 0.12, 95% CI: [0.11−0.15]. Without lack of generality we focus on the case η > 0 in our analytical treatment, then we consider the range 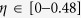 for the numerical solutions of the system’s equations and 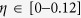 for Monte Carlo simulations. The case *η* = 0 corresponds to the classic SIR model with frequency-dependent transmission rate while *η* = 1 corresponds to the density-dependent case. The immediate consequence of the scaling of contacts is that the basic reproductive number *R*_0_, i.e. the average number of newly infected individuals generated by an infectious one in a fully susceptible population^36^, depends on the population size as (see Material and Methods for the complete derivation):


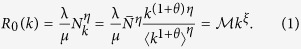


In this expression 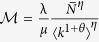 is a constant that depends on the characteristics of the disease and the metapopulation structure. It is immediate to see from Eq. 1 that *R*_0_(*k*) will significantly vary from one location to another, depending on the degree of each node and on the exponent 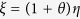, which combines the heterogeneity of the traffic flows and of the contact rates.

The necessary and sufficient condition for the local spreading of the disease in nodes of degree *k* is given by the local epidemic threshold, i.e. 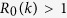. It is important to notice that this may not be satisfied in all the subpopulations. Such situation is realistic for a number of epidemic scenarios where, due to specific characteristics of the local population, the value of the basic reproductive number varies across locations^41^. The crucial question in metapopulations systems is evaluating the conditions under which a local epidemic outbreak leads to a global outbreak. This implies defining an invasion threshold R_*_ for the whole system^17^. In order to find an analytical expression for R_*_, we describe the epidemic invasion as a branching process^11^,^14^,^15^,^17^,^42^ relating the number of subpopulations of degree *k* that have been reached by the epidemic at generation *n*, 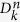, with 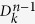:


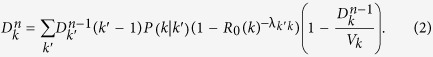


The term 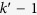 considers that each diseased subpopulation of degree 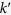 and generation *n* − 1, 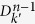, can seed all the connected nodes but the one from which it received the infection. The term 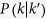 describes the probability that nodes of degree 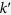 are connected with nodes of degree *k*. We consider uncorrelated networks where this conditional probability does not depend on 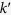 and 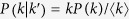. The term 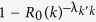 defines the probability that, given 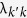 infectious individuals seeding a node of degree *k*, the subpopulation will experience a local outbreak^43^. This number can be estimated as:


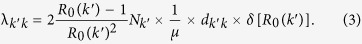


Indeed, the total number of infected individuals generated at the source can be approximated as 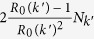
17: infectious individuals recover, on average, after *μ*^−1^ time steps, and the diffusion rate between the two degree classes is 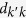. It is important to notice that such approximations are valid only for 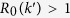. Indeed, if this condition is not satisfied the disease will not be able to spread locally in any subpopulation 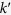. To address this issue, we introduce a step function:


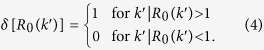


Finally, the last term in Eq. 2 represents the fraction of subpopulations of degree *k* that are not yet infected. By plugging all these terms in Eq. 2, it is possible to solve it analytically and find an explicit expression for the global epidemic threshold:


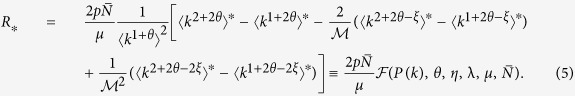


See Material and Methods for more details and the Supplementary Information file for the complete derivation. All the moments denoted by a star are calculated over a subset of degree values. More specifically, we define the general starred degree moment as 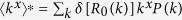. The function 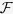 describes the dependence of the threshold on the properties of the network, the mobility patterns, the scaling of contacts, and the details of the disease. Interestingly, the denominator factor 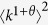 is related to the mobility between subpopulations and not to the spreading dynamics within nodes, therefore the corresponding moment of the degree distribution is calculated over all the values of *k*.

The expression of the global invasion threshold defines the range of parameters for which a global outbreak is possible, corresponding to the solutions of the equation R_*_ = 1. For R_*_ < 1 an outbreak seeded in any subpopulation will eventually die, while for R_*_ > 1 the contagion process will eventually reach a finite fraction of the system with non-zero probability. The transition between the two regimes is typically continuous^15^. In order to isolate the effects introduced by heterogeneous contact rates, we study the system dynamics for different values of *η* compared to the case *η* = 0 that has been previously studied^15^,^17^. Indeed, from Eq. 5 it is possible to see that, by setting *η* = *ξ* = 0, we consistently recover the same expression of R_*_ derived in the case of a constant contact rate across subpopulations^17^. In particular, we compare the value of the critical mobility rate *p*_*c*_, corresponding to the solution of R_*_ = 1 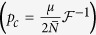, in the two cases: *η* > 0 and *η* = 0. The introduction of a scaling contact rate in every subpopulation, modifies the result of ref. 17 by increasing the overall heterogeneity of the metapopulation system and, eventually, by reducing the critical value of *p*. More specifically, values of *η* > 0 as observed from empirical social networks, alter the spreading dynamics by accelerating the contagion process and thus increasing the value of R_*_. This implies that, for a given set of parameters describing the mobility network, the metapopulation system and the transmissibility of the infectious agent, the critical mobility value will be lower for larger values of *η*. Figure 1A shows the invasion region in the plane 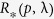 for *η* = 0 and *η* = 0.12, with the latter clearly displaying a larger portion of the phase space in the global spreading regime. In particular, the scaling of contacts with subpopulation sizes allows the global spreading of diseases characterized by significantly smaller values of transmissibility λ. As η increases, the difference in the mobility threshold p_*c*_ grows smaller with constant λ, while the critical transmissibility λ_*c*_ decreases continuously for a given value of p (see Fig. S5 of the Supplementary Information file).

**Figure 2:**
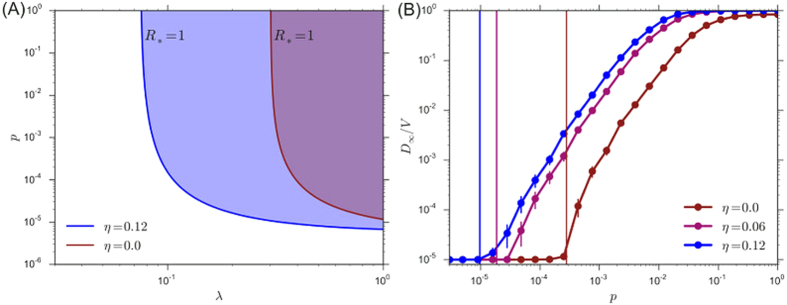
(**A**) Phase diagram defined by the threshold condition R_*_(*p*, λ) = 1, corresponding to the solid lines, for *η* = 0 and *η* = 0.12. We consider uncorrelated scale-free networks of *V* = 10^5^ nodes, and *P*(*k*) ~ *k*^−2.1^. We set *θ* = 0.5, 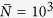, and *μ* = 0.3. (**B**) Simulated global attack rate *D*_∞_/*V* as a function of the mobility rate *p* for different values of the contact scaling exponent *η* = 0, 0.06, 0.12 and λ = 0.35. Vertical lines indicate the critical threshold value *p*_*c*_ calculated by setting R_*_ = 1 in Eq. 5. Each point is averaged over at least 2 × 10^3^ simulations. Error bars correspond to the standard error of the mean.

We confirm our analytical findings through extensive numerical simulations performed considering uncorrelated scale-free networks with *V* = 10^5^ nodes, and exponent *γ* = 2.1 44. In Fig. 2B, we compare the global attack rate, i.e. the final fraction of subpopulations that experienced a local outbreak, for two identical metapopulation structures and different values of *η* (*η* = 0.06, 0.12). The results of 2 × 10^3^ Monte Carlo simulations per point show a very good agreement with the theoretical threshold calculated from Eq. 5.

**Figure 3:**
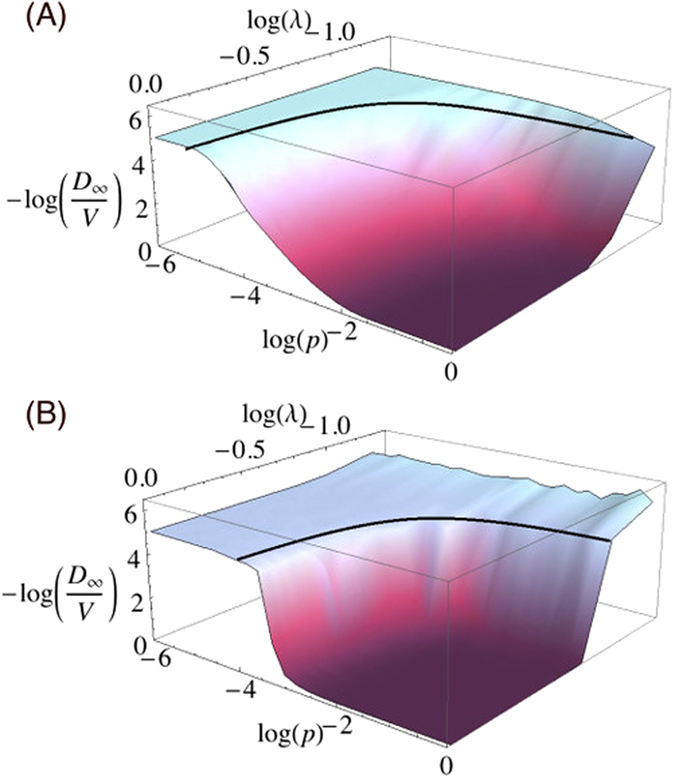
Simulated global attack rate *D*_∞_/*V* as a two-dimensional function of the mobility rate *p* and the transmissibility λ for different mobility network structures characterized by *θ* = 0.5 (A) and *θ* = −0.4 (B). Black solid lines indicate the analytical predictions for the critical values of *p* and λ corresponding to R_*_ = 1. Here the network parameters are the same as in Fig. 2 and *η* = 0.12. Each point of the phase-space is averaged over 2 × 10^3^ simulations. To facilitate the visual comparison between the simulations and the analytical solutions we plot the *z*-axis considering the negative log_10_ of *D*_∞_/*V*.

Overall, the global epidemic threshold is determined in a non-linear way, through the exponent *ξ*, by the interplay between the contact rate heterogeneity, tuned by the exponent *η*, and the heterogeneity of the mobility patterns, tuned by the exponent *θ*. The latter can be changed to counterbalance the effect of the contact scaling on the spreading process. In Fig. 3, we show that considering uncorrelated scale-free networks and constant *η* = 0.12, higher values of *θ* correspond to a lower critical mobility rate and a larger invasion regime phase space. On the other side, by assuming a negative value of *θ*, thus a more homogeneous distribution of the mobility flows across the network, the global spreading regime is suppressed. In both cases, it is remarkable that the numerical simulations show a very good agreement with the theoretical value of the threshold (black solid line in Fig. 3), on the full (*p* − λ) parameter space. Also in this case we considered uncorrelated scale-free networks with *V* = 10^5^ nodes, and exponent *γ* = 2.1. Each point is averaged in 2 × 10^3^ Monte Carlo simulations. In the Methods Section, we report the full details of the numerical simulations.

It is worth to notice that the value of the global threshold R_*_, and, by extension, the value of p_*c*_*, depends explicitly on the size of the system, through the moments of the degree distribution of the network. In the regime of large network size and for a range of parameters γ, θ and η that encloses those measured in real systems, it is possible to show that the critical mobility value p*_*c*_ scales as 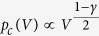 (see the Supplementary Information file for the full derivation). Interestingly, the threshold vanishes for 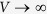, and its trend explicitly depends only on the exponent γ regulating the heterogeneity of the metapopulation network. Beside the dependence of the global threshold on the network size, our analytical treatment is based on a number of assumptions that may not be satisfied in small size systems. Consequently, by reducing the network size we might expect the numerical invasion threshold to deviate from our theoretical predictions. To test the limits of our treatment, we performed numerical simulations on networks of decreasing size (*V* = 10^4^ *and V* = 10^3^). Simulation results, shown in Fig. S8 of the Supplementary Information file, indicate that the theoretical predictions are accurate down to the size V = 10^3^, where finite size effects become more evident.

## Discussion

In the present work, prompted by empirical findings, we derived a general framework to study spreading processes in metapopulation systems where the individual contact rates scale with subpopulation sizes.

The scaling properties of social interactions have been derived here and elsewhere^31^ from on-line sources and telecommunication datasets. It is not straightforward to assume that such properties would be observed by the analysis of a large-scale contact network of physical interactions. To date, empirical measures of physical contact networks have been limited to relatively small samples of individuals which do not allow to directly test the scaling hypothesis^26^. There is, however, evidence that a number of properties observed in on-line and telecommunication social networks can be mapped onto the corresponding physical contact network. The correlation between networks inferred by communication platforms and face-to-face interactions has been recently measured in mobile phone data^45^,^46^. Also, a recent study of contact networks between high-school students found that 67% of the links of their face-to-face contact network, measured with proximity sensors, is present in their Facebook network^47^. Moreover, links of the face-to-face network with aggregate duration larger than a certain threshold correspond all to contacts between Facebook friends^47^. In the case of Twitter, a similar direct empirical comparison is still missing, but recent studies have shown that Twitter mentions reproduce relevant features of real social and mobility networks^30^,^48^,^49^,^50^,^51^. For example, on Twitter individuals devote the large fraction of their communications to a small fraction of ties, i.e. strong ties, and the remaining to occasional contacts, i.e. weak ties^49^. Furthermore, strong ties are statistically localized within the same city^48^,^51^*. Overall, such observations provide indirect evidence supporting the use of Twitter data as a proxy of real social ties relevant for contagion processes. Eventually, it will be important to further confirm the scaling behavior of social contacts by the analysis of additional on-line datasets and, where possible, using real large-scale contact networks.*

The effects of local properties of the subpopulations in RD processes, including different local mixing patterns, have been studied in previous works^52^,^53^,^54^,^55^,^56^,^57^,^58^, but they were generally limited to simplified assumptions on the local contact structure, such as considering only two different contact rates^54^,^57^,^58^, and by always assuming a constant diffusion rate^53^,^56^,^58^. Some recent papers have also considered a power-law distribution of the infectious rates in a metapopulation model^53^,^59^. However, a comprehensive framework that takes into account the interplay between the heterogeneities of both mobility flows and contact rates was still missing. We have shown that the heterogeneity of the contact rates, introduced by the scaling behavior, promotes the epidemic spreading and such effect is enhanced when the distribution of the mobility flows between subpopulations is heterogeneous, as observed in real mobility networks. Our results represent the first step towards a better analytical understanding of contagion processes in structured subpopulations. The proposed framework can be also extended to include behavioral changes, at the population level, triggered by concerns of infection that might induce a reduction in the contact rates.

## Material and Methods

### Reciprocal mention interaction (RMI) network of Twitter users

The Twitter dataset was obtained from the raw Twitter Gardenhose feed^60^. The Gardenhose is an unbiased sampling of about 10% of all tweets from Twitter. We considered only Twitter users that were active during an observation period of 8 months, from January 01 2014 to August 31 2014, and built a reciprocal mention interaction (RMI) network between pairs of users, defined as follows: a link is placed between users A and B if and only if user A mentioned user B in one of his/her tweets and user B mention user A back during the observation period.

### Geographical mapping of Twitter users

A few percentage of the tweets available through the Gardenhose is provided with GPS information. Based on GPS coordinates, we map a tweet into a geographical area using the following procedure. Typically, an active Twitter user would have a sequence of tweets with GPS information within a given time window. We generate the sequence of locations visited by every Twitter user of our dataset, then we mapped every Twitter user into one geographical area based on his/her most frequently visited location – but only if this represented more than 50% of all locations. For example, let’s imagine the Twitter user *i* tweeted sequentially in city A, A, A, B, A, C, A, A, A and D. In this case, city A has the highest probability of appearance 
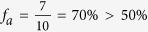
. We therefore mapped user i into city A and call user i a geo-mappable user. In a different case, a user *j* tweeted sequentially in city A, A, B, C, D. Although city A has the highest frequency among the locations visited, the relative probability is 
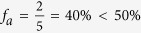
 and no city really dominates the geographic distribution of locations visited by user *j*, thus we do not consider *j* as a geo-mappable user.

In our work, we mapped the Twitter users into two different types of geographical aggregations: the metropolitan areas of United States and Europe combined (referred to as “metropolitan areas”) and the geographical census areas centered around IATA airports^13^ (referred to as “basins”). The United States metropolitan areas are defined by the year 2014 United States urban area/urban cluster shapefile from the TIGER/Line database^37^. The European metropolitan areas are defined by the year 2000 morphological zones shapefile (with population larger than 50000). The shapefile can be obtained from the European Union Open Data Portal^38^.

The geographical census areas centered around IATA airports are defined^13^ by assigning cell of 15′ * 15′ to the closest airport within the same country. The assigning procedure follows a Voronoi-like tessellation^61^ with a cut-off scale for the tassels size of 200 km^2^. After the geo-mapping process, we obtain two subset of users: metropolitan mappable users (MMU) in which each user can be assigned to a metropolitan area in United States or Europe as defined above, or basin mappable users (BMU) in which each user can be assigned to a basin as defined above.

For a given basin/metropolitan area, N is the population, S is the total number of geo-mappable users within the area. The total number of Twitter interactions is 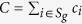, where *c*_*i*_ is the degree of user *i* in the subgraph *S*_*g*_ of the entire RMI network. In one case, we compute *c*_*i*_ as the number of interactions of user *i* confined within the boundary of basin/metropolitan area in the other case, *c*_*i*_ is assumed to be the degree of user *i* in the entire RMI network. Since the coverage of Twitter user *s* = *S*/*N* differs from one basin/metropolitan area to another, the volume of interaction *C*_*r*_ is rescaled as 
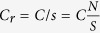
.

### Introducing the scaling of contacts into the SIR model

The SIR is a compartmental model which describes the evolution of a contagious disease in a closed population. The three compartments 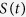, 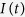, 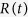 represent respectively the number of susceptible, infectious and recovered people and the total population 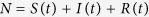 is constant over time.

We assume homogeneous mixing in the population, which means everyone interacts with equal probability with everyone else. Usually it is assumed that the average number of contacts *c* per individual and unit of time is a constant, *c* = *c*_0_ and does not depend on the population size, then, every susceptible individual has *c*_0_ contacts per unit of time and we define *g* as the probability of successful disease transmission following a contact. Therefore is convenient to define the transmission rate λ of the disease as^2^:


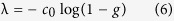


In this framework, it is easy to show that an outbreak of the disease can occur only if 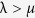, where *μ* the recovery rate of the disease. It is a common practice to define the Basic Reproductive Number of the disease as 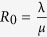, which has to be greater than 1 for the probability of an outbreak to be larger than 0. In our work, we assumed that the total number of contacts 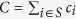 scales super linearly with the population size N:


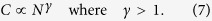


So the average per capita contacts rate can be defined as:


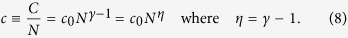


Consequently the transmission rate will be a function of N:


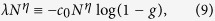


thus also *R*_0_ will be a function of N:


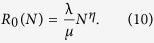


### Branching process

Equation 2 describes the infection dynamics at the level of subpopulations, as a Levins-type process. It can be solved by introducing three main assumptions: the network substrate is uncorrelated, i.e. 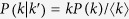, at the early stage of the epidemic the probability that a subpopulation is not already seeded is almost one, i.e. 
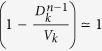
, and, finally, that 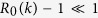
17.

In this case, Eq. 2 can be written as:


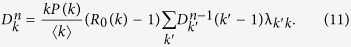


In our framework, it is worth to notice that *R*_0_(*k*) may be smaller than for some values of *k*. Therefore, it is reasonable to assume that in a subpopulation with *R*_0_(*k*) < 1 the probability for an outbreak to occur is zero and its contribution to the number of traveling infected individuals, 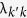, will be zero. This translates into defining 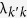 as:


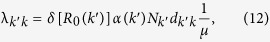


where 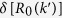 is the Heaviside function of Eq. 4 and the quantity 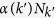 denotes the total number of infected individuals during the evolution of the epidemic in subpopulation 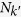. The value of 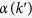 is dependent on the details of the disease, in particular in the case of 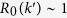:


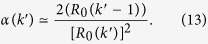


thus leading to the definition of Eq. 3. By recalling the definition of the diffusion rate, 
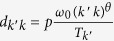
, and the stationary value of *N*_*k*_ at equilibrium, 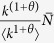, we eventually can write Eq. 11 as:


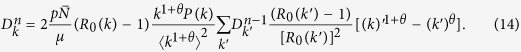


### Invasion threshold

To solve the recursive Eq. 14, it is convenient to define the auxiliary function Θ^*n*^ as:


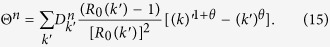


In this way Eq. 11 can be conveniently written in the iterative form:


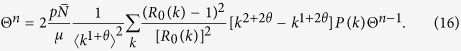


By expanding the term in 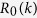:


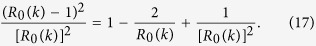


and plugging the explicit form 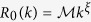, with 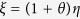, we have:


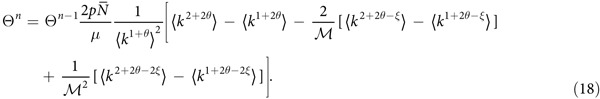


From this equation it is immediate to define the the Global Invasion Threshold R_*_ as described by Eq. 5 and find the threshold condition on the mobility rate:


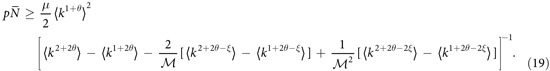


### Simulation Methods

In all our simulations we consider uncorrelated scale-free networks generated with the UCM algorithm^62^. In particular, we study networks formed by 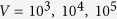 nodes, and degree distribution 
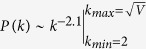
. Each simulation is started by assigning to each subpopulation the population value at equilibrium *N*_*k*_, while keeping the average over the whole system constant, 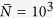. Furthermore, we seed a randomly selected subpopulation among those with 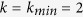 with 10% of infected individuals. We run the spreading process until the number of infected individuals in the system reaches 0. We consider as diseased any subpopulation in which we observed at least a secondary infection, i.e. an infected seed generates another infected individual. Finally, each simulation point is averaged over 2 × 10^3^ independent simulations. In order to avoid biases associated to specific network structures each simulation is run over a randomly selected network over 60 independent realizations of the UCM algorithm.

## Additional Information

**How to cite this article**: Tizzoni, M. *et al.* The Scaling of Human Contacts and Epidemic Processes in Metapopulation Networks. *Sci. Rep.* **5**, 15111; doi: 10.1038/srep15111 (2015).

## Supplementary Material

Supplementary Information

## Figures and Tables

**Table 1:** Summary of the scaling exponents *γ* measured on the Twitter dataset.

Geographical aggregation	Scaling exponent *γ*
Basins (internal connections only)	1.11 ± 0.01
Basins (all connections)	1.11 ± 0.01
Metro areas (internal connections only)	1.20 ± 0.02
Metro areas (all connections)	1.08 ± 0.02
Basins in US (internal connections only)	1.15 ± 0.01
Basins in US (all connections)	1.16 ± 0.02
Metro areas in US (internal connections only)	1.16 ± 0.02
Metro areas in US (all connections)	1.09 ± 0.03
Basins in Europe (internal connections only)	1.21 ± 0.04
Basins in Europe (all connections)	1.06 ± 0.01
Metro areas in Europe (internal connections only)	1.18 ± 0.02
Metro areas in Europe (all connections)	1.08 ± 0.02

Error intervals correspond to the standard error of the slope in the regression fit. When referring to Europe, the following 31 countries are taken into consideration: Belgium, France, Bulgaria, Bosnia Herzegovina, Croatia, Germany, Hungary, Finland, Denmark, Netherlands, Portugal, Latvia, Lithuania, Luxembourg, Romania, Poland, Greece, Estonia, Italy, Albania, Czech Republic, Cyprus, Austria, Ireland, Spain, Macedonia, Slovakia, Malta, Slovenia, United Kingdom, Sweden.
