# Supplementary material for: The Scaling of Human Contacts and Epidemic Processes in Metapopulation Networks
Source: Sci Rep. 2015 Oct 19;5:15111. doi: 10.1038/srep15111 (PMC4609962; doi:10.1038/srep15111)
Supplement: Supplementary Information [file srep15111-s1.pdf]

Supplementary Information for *The Scaling of Human Contacts and Epidemic Processes in Metapopulation Networks*

Michele Tizzoni,[1,*] Kaiyuan Sun,[2,†] Diego Benusiglio,[1,3,‡] Márton Karsai,[4,§] and Nicola Perra[2,¶]

[1] *Computational Epidemiology Laboratory, ISI Foundation, Torino, via Alassio 11/C, Italy*
[2] *Laboratory for the Modeling of Biological and Socio-technical Systems, Northeastern University, Boston MA 02115 USA*
[3] *Dipartimento di Fisica, Universitá degli Studi di Torino, via Giuria 1, Torino, Italy*
[4] *Laboratoire de l'Informatique du Parallélisme, INRIA-UMR 5668, IXXI, ENS de Lyon, 69364 Lyon, France*
(Dated: July 14, 2015)

ESTIMATING THE SCALING EXPONENT OF CONTACTS FROM TWITTER DATA

To test the super-linear scaling hypothesis on our Twitter dataset, we adopt the following statistical method. We fit the rescaled cumulative degree C_r to a power-law function of the population of the census area, both normalized by their averages, using a linear regression of the log-transformed variables, in the form $\ln(C_r/\langle C_r\rangle) = \gamma \ln(N/\langle N\rangle) + \epsilon$, and compare the result against a null model, represented by a linear function of the rescaled population, $\ln(C_r/\langle C_r\rangle) = \ln(N/\langle N\rangle) + \hat{\epsilon}$, where ϵ and $\hat{\epsilon}$ are constant.

We quantify the goodness of fit by computing the adjusted R^2 for both models. We then compare the results of the regression by using a Student's t-test to quantify the significance of the difference between the two slopes. More specifically, under the null hypothesis that there is no difference between linear and super-linear models, we compute the associated Z-score: $Z = \frac{|\gamma-1|}{\sigma_\gamma}$, where σ_γ is the standard error of the slope γ. If the sample size is s, then the p-value associated to Z can be obtained from the survival function of Student's t distribution: $p = t(Z, s-2)$.

Figures S1 and S2 show the comparison between the super-linear model and the linear one, for all the datasets under study. In all cases, the super-linear function is a better fit to the data than the linear one (R^2 is always larger for the super-linear fit) and the difference between the slopes is always statistically significant, with $p < 0.001$.

SCALING OF INTERACTIONS BEYOND THE BOUNDARY OF GEOGRAPHICAL AREAS

For a given basin/metropolitan area, N is the population, S is the total number of geo-mappable users within the area. The total number of Twitter interactions $C = \sum_{i\in S} c_i$. Different from the above case, c_i is assumed to be the degree of user i in the entire RMI network. In this case, c_i is no longer confined within the basin/metropolitan area boundary and the interactions between user i and users from other basin/metropolitan areas or users that are not geo-mappable are also taken into account. The volume of interactions C_r is rescaled as $C_r = C/s = C\frac{N}{S}$. As shown in Figure S2, for the case of BMU we find $\gamma = 1.11 \pm 0.01$ in the US, $\gamma = 1.06 \pm 0.01$ in Europe [4] and $\gamma = 1.11 \pm 0.01$ when considering all the basins in the world. For the case of MMU, we find $\gamma = 1.07 \pm 0.01$ in the US, $\gamma = 1.09 \pm 0.03$ in Europe and $\gamma = 1.08 \pm 0.02$ when combining all the metropolitan areas of the US and Europe together. See Table S1.

COMPLETE DERIVATION OF THE GLOBAL INVASION THRESHOLD

SIR is a compartmental model which describes the evolution of a contagious disease in a closed population. The three compartments $S(t)$, $I(t)$, $R(t)$ represent respectively the number of susceptible, infectious and recovered people. The total population $N = S(t) + I(t) + R(t)$ is constant, so we can define the fraction of people in each compartment simply dividing by N:

$$s(t) = \frac{S(t)}{N};\ i(t) = \frac{I(t)}{N};\ r(t) = \frac{R(t)}{N} \quad (1)$$

so that $s(t) + i(t) + r(t) = 1,\ \forall t$.

Here, we derive the expression of $R_0(k)$ including the scaling of human contacts with subpopulation sizes. For clarity in the exposition let us start from the derivation of the reproductive number in the case within the frequent-dependent approximation.

Geographical aggregation	Scaling exponent γ
Basins (internal connections only)	1.11 ± 0.01
Basins (all connections)	1.11 ± 0.01
Metro areas (internal connections only)	1.20 ± 0.02
Metro areas (all connections)	1.08 ± 0.02
Basins in US (internal connections only)	1.15 ± 0.01
Basins in US (all connections)	1.16 ± 0.02
Metro areas in US (internal connections only)	1.16 ± 0.02
Metro areas in US (all connections)	1.09 ± 0.03
Basins in Europe (internal connections only)	1.21 ± 0.04
Basins in Europe (all connections)	1.06 ± 0.01
Metro areas in Europe (internal connections only)	1.18 ± 0.02
Metro areas in Europe (all connections)	1.08 ± 0.02

TABLE S1: Summary of the scaling exponents γ measured on the Twitter dataset. Error intervals are given by the standard error of the slope in the regression fit.

R_0 in SIR models

Let us assume homogeneous mixing in the population, which means everyone interacts with equal probability with everyone else. Every susceptible individual has c_0 contacts per unit of time and we define g as the probability of successful disease transmission following a contact.

Therefore is convenient to define the transmission rate λ of the disease as [1]:

$$\lambda = -c_0 \; log(1-g) \tag{2}$$

and μ the recovery rate of the disease.

The dynamics of the model is described by the following system of equations:

$$\frac{ds(t)}{dt} = -\lambda \; i(t)s(t) \tag{3}$$

$$\frac{di(t)}{dt} = \lambda \; i(t)s(t) - \mu i(t) \tag{4}$$

$$\frac{dr(t)}{dt} = \mu i(t). \tag{5}$$

We are interested in the time evolution of number of infectious people:

$$\frac{di(t)}{dt} = \lambda \; i(t)s(t) - \mu i(t) = i(t)(\lambda s(t) - \mu). \tag{6}$$

In early stage of epidemics the number of susceptible people is almost the whole population $s(i) \simeq 1$, so:

$$\frac{di(t)}{dt} \simeq i(t)(\lambda - \mu). \tag{7}$$

Since $(\lambda - \mu)$ does not change over time we can integrate obtaining:

$$i(t) \simeq i(t_0) \; e^{(\lambda-\mu)t}. \tag{8}$$

We conclude that an outbreak of the disease can occur only if $\lambda > \mu$. It is a common practice to define the Basic Reproductive Rate of the disease:

$$R_0 = \frac{\lambda}{\mu}, \tag{9}$$

which has to be greater than 1 in order to have an outbreak.

Introducing the scaling of contacts

Usually it is assumed that the average number of contacts c per individual and unit of time is a constant and does not depend on the population size. However, recent studies on urban data [2] as well as our observation reported in this paper suggest that the total number of contacts $C = \sum_{i \in S} c_i$ scale super linearly with the population size N:

$$C \propto N^{\gamma} \qquad \text{where} \quad \gamma > 1. \tag{10}$$

So the average per capita contacts rate can be defined as:

$$c \equiv \frac{C}{N} = c_0 N^{\gamma-1} = c_0 N^{\eta} \quad \text{where} \quad \eta = \gamma - 1. \tag{11}$$

Consequently the transmission rate will be a function of N:

$$\lambda N^{\eta} \equiv -c_0 N^{\eta} \log{(1-g)}, \tag{12}$$

where $\lambda \equiv -c_0 log(1-g) > 0$ because $0 < g < 1$ is a probability, thus also R_0 will be a function of N:

$$R_0(N) = \frac{\lambda}{\mu} N^{\eta}. \tag{13}$$

Global invasion threshold in metapopulation networks

Let us consider a metapopulation model defined on a network in which nodes represents spatial regions with a certain population (called *subpopulation*), links represents human mobility patterns between those regions and weights on links quantify the average number of people traveling on each pattern.
The whole network has V nodes identified by a index $i = 1, ..., V$ and the degree distribution of the nodes is $P(k)$. Since we consider network with a heterogeneous degree distribution, a more convenient representation of the systems is provided by the quantities defined in terms of the degree k:

$$N_k = \frac{1}{V_k} \sum_{i|k_i=k} N_i, \tag{14}$$

where V_k is the number of nodes with degree k and the sum run over all nodes i having degree k_i equal to k. Using this degree-block description we can make explicit the dependency of $R_0(N_i)$ from k:

$$R_0(N_i) \longrightarrow R_0(k) \equiv \frac{\lambda}{\mu} N_k^{\eta}. \tag{15}$$

Assuming the scaling of human contacts with population size, the expression of R_0 is a function of the population N_k in each patch:

$$R_0(k) = \frac{\lambda}{\mu} N_k^{\eta}. \tag{16}$$

We introduce this new hypothesis in the basic formula for the evolution of the number D_k^n of *diseased* subpopulation of degree k at epidemic generation n :

$$D_k^n = \sum_{k'} D_{k'}^{n-1} (k'-1) P(k|k') \left[1 - \left(\frac{1}{R_0(k)} \right)^{\lambda_{k'k}} \right] \left(1 - \frac{D_k^{n-1}}{V_k} \right). \tag{17}$$

At early stage of epidemics the probability that the subpopulation is not already seeded by infected individuals is almost one $\left(1 - \frac{D_k^{n-1}}{V_k}\right) \simeq 1$, so:

$$D_k^n = \sum_{k'} D_{k'}^{n-1} (k'-1) P(k|k') \left[1 - \left(\frac{1}{R_0(k)} \right)^{\lambda_{k'k}} \right]. \tag{18}$$

Considering the case of uncorrelated networks, i.e. $P(k|k') = \frac{kP(k)}{\langle k\rangle}$ and that $R_0(k) - 1 << 1$; in this limit we can approximate the local outbreak probability as:

$$\left[1 - \left(\frac{1}{R_0(k)}\right)^{\lambda_{k'k}}\right] \simeq (R_0(k) - 1)\lambda_{k'k}. \tag{19}$$

In this case we obtain:

$$D_k^n = \frac{kP(k)}{\langle k\rangle}(R_0(k) - 1)\sum_{k'} D_{k'}^{n-1}(k' - 1)\lambda_{k'k}. \tag{20}$$

The quantity $\lambda_{k'k}$ can be estimated as:

$$\lambda_{k'k} = d_{k'k}\frac{\alpha(k')}{\mu}N_{k'}. \tag{21}$$

In the case of diffusion proportional to the product of the degrees:

$$d_{k'k} = p\,\frac{\omega_0(k'k)^\theta}{T_{k'}}, \tag{22}$$

where p is the diffusion rate and the term $T_{k'}$ in the traffic depend mobility case is defined as:

$$T_{k'} = k'\sum_k P(k|k')\omega_0(k'k)^\theta = \omega_0\frac{\langle k^{1+\theta}\rangle}{\langle k\rangle}(k')^{1+\theta}, \tag{23}$$

thus,

$$d_{k'k} = p\,\frac{\langle k\rangle}{\langle k^{1+\theta}\rangle}\frac{k^\theta}{k'}, \tag{24}$$

and

$$\lambda_{k'k} = p\frac{\langle k\rangle}{\langle k^{1+\theta}\rangle}\frac{(k)^\theta}{k'}\frac{\alpha(k')}{\mu}N_{k'}. \tag{25}$$

The stationary solution $\partial_t N_k(t) = 0$ of the equation that describes the dynamics of individual, gives the population in a node of degree k in the stationary state:

$$N_k = \frac{k^{(1+\theta)}}{\langle k^{1+\theta}\rangle}\overline{N} \quad \text{where} \quad \overline{N} = \sum_k P(k)N_k, \tag{26}$$

then

$$\lambda_{k'k} = p\frac{\langle k\rangle}{\langle k^{1+\theta}\rangle^2}\frac{\alpha(k')}{\mu}(k'k)^\theta\,\overline{N}. \tag{27}$$

Substituting in the expression for D_k^n :

$$D_k^n = (R_0(k) - 1)\frac{k^{1+\theta}P(k)}{\langle k^{1+\theta}\rangle^2}\frac{p\overline{N}}{\mu}\sum_{k'}\alpha(k')\;D_{k'}^{n-1}k'^\theta(k' - 1). \tag{28}$$

In the case of macroscopic outbreak, in a closed population, the total number of infected individuals during the evolution of the epidemic will be equal to $\alpha(k)N_k$. The value of $\alpha(k)$ is dependent on the details of the disease, in particular in the case of $R_0 \sim 1$:

$$\alpha(k) \simeq \frac{2(R_0(k) - 1)}{[R_0(k)]^2}. \tag{29}$$

So we get:

$$D_k^n = 2\frac{p\overline{N}}{\mu}(R_0(k)-1)\frac{k^{1+\theta}P(k)}{\langle k^{1+\theta}\rangle^2}\sum_{k'} D_{k'}^{n-1}\frac{(R_0(k')-1)}{[R_0(k')]^2}\ [(k)'^{1+\theta}-(k')^{\theta}]. \tag{30}$$

Let us define the auxiliary function Θ^{n-1} as:

$$\Theta^{n-1} = \sum_{k'} D_{k'}^{n-1}\frac{(R_0(k')-1)}{[R_0(k')]^2}\ [(k)'^{1+\theta}-(k')^{\theta}]. \tag{31}$$

We can rewrite the expression for D_k^n using the auxiliary function obtaining:

$$D_k^n = 2\frac{p\overline{N}}{\mu}(R_0(k)-1)\frac{k^{1+\theta}P(k)}{\langle k^{1+\theta}\rangle^2}\Theta^{n-1}. \tag{32}$$

So the expression for the auxiliary function can be conveniently written in the iterative form:

$$\Theta^n = 2\frac{p\overline{N}}{\mu}\frac{1}{\langle k^{1+\theta}\rangle^2}\sum_{k'}\frac{(R_0(k')-1)^2}{[R_0(k')]^2}[(k')^{2+2\theta}-(k')^{1+2\theta}]P(k')\Theta^{n-1}. \tag{33}$$

In the sum over k' there are no terms that depend on k so:

$$\Theta^n = 2\frac{p\overline{N}}{\mu}\frac{1}{\langle k^{1+\theta}\rangle^2}\sum_{k}\frac{(R_0(k)-1)^2}{[R_0(k)]^2}[k^{2+2\theta}-k^{1+2\theta}]P(k)\Theta^{n-1}. \tag{34}$$

We can write:

$$\frac{(R_0(k)-1)^2}{[R_0(k)]^2} = 1-\frac{2}{R_0(k)}+\frac{1}{[R_0(k)]^2}. \tag{35}$$

Then:

$$\Theta^n = \Theta^{n-1}\frac{2p\overline{N}}{\mu}\frac{1}{\langle k^{1+\theta}\rangle^2}\left[\sum_k[k^{2+2\theta}-k^{1+2\theta}]P(k)-2\sum_k[k^{2+2\theta}-k^{1+2\theta}]\frac{1}{R_0(k)}P(k)+\sum_k[k^{2+2\theta}-k^{1+2\theta}]\frac{1}{[R_0(k)]^2}P(k)\right]. \tag{36}$$

As we said before, the value of $R_0(k)$ is:

$$R_0(k) = \frac{\lambda}{\mu}N_k^{\eta} = \frac{\lambda}{\mu}\left(\frac{k^{1+\theta}}{\langle k^{1+\theta}\rangle}\overline{N}\right)^{\eta} = \mathcal{M}k^{\xi}, \tag{37}$$

where $\mathcal{M} = \frac{\lambda}{\mu}\frac{\overline{N}^{\eta}}{\langle k^{1+\theta}\rangle^{\eta}}$ and $\xi = (1+\theta)\eta$. Plugging this in the previous equation we have:

$$\Theta^n = \Theta^{n-1}\frac{2p\overline{N}}{\mu}\frac{1}{\langle k^{1+\theta}\rangle^2}\left[\langle k^{2+2\theta}\rangle-\langle k^{1+2\theta}\rangle-\frac{2}{\mathcal{M}}\sum_k[k^{2+2\theta-\xi}-k^{1+2\theta-\xi}]P(k)+\frac{1}{\mathcal{M}^2}\sum_k[k^{2+2\theta-2\xi}-k^{1+2\theta-2\xi}]P(k)\right]. \tag{38}$$

That yield to:

$$\Theta^n = \Theta^{n-1}\frac{2p\overline{N}}{\mu}\frac{1}{\langle k^{1+\theta}\rangle^2}\left[\langle k^{2+2\theta}\rangle-\langle k^{1+2\theta}\rangle-\frac{2}{\mathcal{M}}[\langle k^{2+2\theta-\xi}\rangle-\langle k^{1+2\theta-\xi}\rangle]++\frac{1}{\mathcal{M}^2}[\langle k^{2+2\theta-2\xi}\rangle-\langle k^{1+2\theta-2\xi}\rangle]\right]. \tag{39}$$

It is natural to define the Global Invasion Threshold R_* as:

$$R_* = \frac{2p\overline{N}}{\mu}\frac{1}{\langle k^{1+\theta}\rangle^2}\left[\langle k^{2+2\theta}\rangle-\langle k^{1+2\theta}\rangle-\frac{2}{\mathcal{M}}[\langle k^{2+2\theta-\xi}\rangle-\langle k^{1+2\theta-\xi}\rangle]+\frac{1}{\mathcal{M}^2}[\langle k^{2+2\theta-2\xi}\rangle-\langle k^{1+2\theta-2\xi}\rangle]\right], \tag{40}$$

that allows the increasing of infected subpopulations and a global epidemic in the metapopulation process only if

$$R_* > 1. \tag{41}$$

So we immediately find the condition on the mobility rate:

$$p\overline{N} \geq \frac{\mu}{2}\langle k^{1+\theta}\rangle^2\left[\langle k^{2+2\theta}\rangle-\langle k^{1+2\theta}\rangle-\frac{2}{\mathcal{M}}[\langle k^{2+2\theta-\xi}\rangle-\langle k^{1+2\theta-\xi}\rangle]+\frac{1}{\mathcal{M}^2}[\langle k^{2+2\theta-2\xi}\rangle-\langle k^{1+2\theta-2\xi}\rangle]\right]^{-1}. \tag{42}$$

General solution accounting for the $R_0(k) < 1$ cases

Introducing the scaling of contacts, it might happen that $R_0(k) < 1$ for some degree classes (see Figure S3). Neglecting this condition introduces non physical terms in the threshold as shown in Figure S4 (red line). It is reasonable considering that in subpopulation with $R_0(k) < 1$ the probability for an outbreak to occur is zero:

$$1 - R_0(k)^{-\lambda_{k'k}} = 0, \quad \forall k | R_0(k) < 1. \tag{43}$$

All the expression obtained remain the same with a caveat: not all the k contribute to the moments. In other words the basic expression for D_k^n becomes:

$$D_k^n = \sum_{k'} D_{k'}^{n-1}(k'-1)P(k|k')\left[1 - \left(\frac{1}{R_0(k)}\right)^{\lambda_{k'k}}\right]. \tag{44}$$

Considering, as before, uncorrelated networks and $R_0(k) - 1 << 1$:

$$D_k^n = (R_0(k)-1)\frac{kP(k)}{\langle k\rangle}\sum_{k'} D_{k'}^{n-1}(k'-1)\lambda_{k'k}. \tag{45}$$

And the quantity $\lambda_{k'k}$ become:

$$\lambda_{k'k} = \delta[R_0(k')]\alpha(k')N_{k'}d_{k'k}\frac{1}{\mu}, \tag{46}$$

or using the expression 29 for $\alpha(k')$:

$$\lambda_{k'k} \simeq \delta[R_0(k')]\frac{2(R_0(k')-1)}{R_0(k')^2}N_{k'}d_{k'k}\frac{1}{\mu}, \tag{47}$$

where the δ is the step function:

$$\delta[R_0(k)] = \begin{cases} 1 & \text{for } k \mid R_0(k) > 1 \\ 0 & \text{for } k \mid R_0(k) < 1. \end{cases} \tag{48}$$

Plugging this term in Eq. 45 the expression for the Global Invasion Threshold becomes:

$$R_* = \frac{2p\bar{N}}{\mu}\frac{1}{\langle k^{1+\theta^2}\rangle}\left[\langle k^{2+2\theta}\rangle^* - \langle k^{1+2\theta}\rangle^* - \frac{2}{\mathcal{M}}\left[\langle k^{2+2\theta-\xi}\rangle^* - \langle k^{1+2\theta-\xi}\rangle^*\right] + \frac{1}{\mathcal{M}^2}\left[\langle k^{2+2\theta-2\xi}\rangle^* - \langle k^{1+2\theta-2\xi}\rangle^*\right]\right], \tag{49}$$

where each moment $\langle k^x\rangle^* = \sum_{k|R_0(k)>1} k^x P(k)$. Since $R_0 = \mathcal{M}k^\xi$, it is an increasing and monotonous function of k. So we can find $k^* \mid \forall k > k^*,\ R_0(k) > 1$. In other words, we can simply define:

$$\langle k^x\rangle^* = \sum_{k>k^*} k^x P(k). \tag{50}$$

Finally the condition on the mobility rate becomes:

$$p\overline{N} \geq \frac{\mu}{2}\langle k^{1+\theta}\rangle^2\left[\langle k^{2+2\theta}\rangle^* - \langle k^{1+2\theta}\rangle^* - \frac{2}{\mathcal{M}}[\langle k^{2+2\theta-\xi}\rangle^* - \langle k^{1+2\theta-\xi}\rangle^*] + \frac{1}{\mathcal{M}^2}[\langle k^{2+2\theta-2\xi}\rangle^* - \langle k^{1+2\theta-2\xi}\rangle^*]\right]. \tag{51}$$

It is important to notice that just the moments in the squared parenthesis are done in a subset of k. The moment outside the parenthesis considers every k as it is related to the mobility and not to the internal spreading dynamics. Clearly the mobility threshold is

$$p_c = \frac{\mu}{2\overline{N}}\langle k^{1+\theta}\rangle^2\left[\langle k^{2+2\theta}\rangle^* - \langle k^{1+2\theta}\rangle^* - \frac{2}{\mathcal{M}}[\langle k^{2+2\theta-\xi}\rangle^* - \langle k^{1+2\theta-\xi}\rangle^*] + \frac{1}{\mathcal{M}^2}[\langle k^{2+2\theta-2\xi}\rangle^* - \langle k^{1+2\theta-2\xi}\rangle^*]\right]. \tag{52}$$

Mobility threshold as a function of η

In the main text we show the phase space $R_*(p, \lambda)$ for $\eta = 0$ and $\eta = 0.12$. Here, we show the behavior of the invasion threshold for a larger set of η. As clear from Fig S5 increasing the value of η results in a larger phase space where the invasion is possible. In particular, it is interesting to notice that different values of η are characterized by similar values of p_c, but very different values of λ_c.

Mobility threshold as a function of V

Real networks have finite size, thus the distributions characterizing their features, as the degree, are defined by an upper bound. In our study, we consider uncorrelated scale-free networks generated with the configuration model [3]. In order to avoid structural correlations, the algorithm sets $k_{max} = \sqrt{V}$. Given such constraint, the invasion threshold is a function of the network size through the moments of the degree distribution appearing in its definition. In this section we derive the explicit dependence. To this end, let us consider the critical value of the mobility rate, p_c, and its dependence on V as:

$$p_c(V) \sim \frac{\mathcal{A}(V)}{\mathcal{B}(V) + \mathcal{C}(V)} \tag{53}$$

where we defined $\mathcal{A}(\mathcal{V}) = \langle k^{1+\theta} \rangle^2$, $\mathcal{B}(V) = \langle k^{2+2\theta} \rangle^* - \langle k^{1+2\theta} \rangle^*$, and $\mathcal{C}(V) = -\frac{2}{\mathcal{M}}[\langle k^{2+2\theta-\xi} \rangle^* - \langle k^{1+2\theta-\xi} \rangle^*] + \frac{1}{\mathcal{M}^2}[\langle k^{2+2\theta-2\xi} \rangle^* - \langle k^{1+2\theta-2\xi} \rangle^*]$

Let us derive the explicit dependence of V for each term, starting with the numerator:

$$\mathcal{A}(\mathcal{V}) = \langle k^{1+\theta} \rangle^2 = \left(\frac{1-\gamma}{V^{\frac{1-\gamma}{2}} - k_{min}^{1-\gamma}} \frac{1}{2+\theta-\gamma} k^{2+\theta-\gamma} |_{k_{min}}^{\sqrt{V}} \right)^2 \tag{54}$$

In the limit of large V, and for any value of γ such that $1 < \gamma < 2 + \theta$, we can write:

$$\mathcal{A}(V) \propto V^{2+\theta-\gamma}. \tag{55}$$

Let us consider the second term:

$$\mathcal{B}(V) = \langle k^{2+2\theta} \rangle^* - \langle k^{1+2\theta} \rangle^* = \frac{1-\gamma}{V^{\frac{1-\gamma}{2}} - k^{*(1-\gamma)}} \left(\frac{1}{3+2\theta-\gamma} k^{3+2\theta-\gamma} |_{k^*}^{\sqrt{V}} - \frac{1}{2+2\theta-\gamma} k^{2+2\theta-\gamma} |_{k^*}^{\sqrt{V}} \right) \tag{56}$$

In the limit of large V, $k^* \ll \sqrt{V}$, and for any value of γ such that $1 < \gamma < 2 + 2\theta$, we can write

$$\mathcal{B}(V) \propto V^{\frac{3+2\theta-\gamma}{2}} \tag{57}$$

Let us now consider the last term

$$\mathcal{C}(V) = -\frac{2}{\mathcal{M}}[\langle k^{2+2\theta-\xi} \rangle^* - \langle k^{1+2\theta-\xi} \rangle^*] + \frac{1}{\mathcal{M}^2}[\langle k^{2+2\theta-2\xi} \rangle^* - \langle k^{1+2\theta-2\xi} \rangle^*]. \tag{58}$$

In the limit of large V, $k^* \ll \sqrt{V}$, and for any value of γ such that $1 < \gamma < 2(1+\theta)(1-\eta)$, we can write

$$\mathcal{C}(V) \propto V^{\frac{3+2\theta-\gamma+2\eta(1-\gamma)}{2}} \tag{59}$$

Interestingly, for any value of $\gamma > 1$ the leading order in the denominator is $\mathcal{B}(V)$. Thus we can write

$$p_c(V) \propto V^{\frac{1-\gamma}{2}} \tag{60}$$

It is important stressing that the validity of such scaling is limited in the regime of large V, $k^* \ll \sqrt{V}$, and $1 < \gamma < \min_{\theta,\eta}[2+\theta, 2+2\theta, 2(1+\theta)(1-\eta)]$. For any general real value of θ and η we could have $\min_{\theta,\eta}[2+\theta, 2+2\theta, 2(1+\theta)(1-\eta)] < 1$. In this case the scaling behavior derived would not be correct. Instead if $\theta > \frac{2\eta-1}{2(1-\eta)}$ or $\eta < \frac{1+2\theta}{2(1+\theta)}$ the minimum value of those quantities is always larger than one. This is the regime where the scaling derived above holds and, interestingly, it coincides with the region of parameters we study. In fact, one of the settings we considered is $\theta = 0.5$ and $\eta = 0.12$. In general for such value of θ the scaling holds as long as $\eta < 2/3$.

In Figure S6 we compare the empirical scaling evaluated through numerical solution of p_c with the analytical trend derived above. We set $\theta = 0.5$, $\gamma = 2.1$, $\lambda = 0.35$, and $\mu = 0.3$. From the plot we confirm that the scaling is not function of η. Also, the fitted behaviors as function of V are in agreement with the theoretical prediction of $p_c \propto V^{-0.55}$. To further substantiate the comparison in Figure S7 we considered $\gamma = 1.5$. Also in this case, we do not observe a dependence of η, and the numerical values are well captured by the theoretical prediction $p_c \propto V^{-0.25}$.

The mathematical construct we proposed in this paper is, in general, valid for $V \to \infty$. Indeed, beside the explicit dependence on V we just derived, we expect others finite size effects. In fact, in small networks some of the approximations we adopted are not strictly valid, and stochastic effects can become dominant. Consequently, by reducing the system size we expect the experimental invasion threshold to deviate from our theoretical predictions. In Figure S8 we show the behavior of D_∞/V as a function of p for different values of V. As clear, from the plot the theoretical estimation of the mobility threshold (vertical lines) is accurate for $V = 10^5$, while it is underestimated for $V = 10^4$, and overestimated for $V = 10^3$. It is important to mention that the analytical conditions on the mobility threshold we derived provides a lower bound for p. Thus empirical thresholds larger than theoretical predictions are still compatible with our analytical results (as for the case $V = 10^4$). Instead, empirical thresholds smaller than theoretical predictions are not compatible with our analytical results (as for the case $V = 10^3$). As expected, decreasing V small finite size effects become increasingly important and deviations from the theoretical predictions become increasingly large.

* michele.tizzoni@isi.it
† k.sun@neu.edu
‡ diego.benusiglio@gmail.com
§ marton.karsai@ens-lyon.fr
¶ n.perra@neu.edu

[1] M. Keeling and P. Rohani, *Modeling Infectious Disease in Humans and Animals* (Princeton University Press, 2008).
[2] M. Schläpfer, L. Bettencourt, S. Grauwin, M. Raschke, R. Claxton, Z. Smoreda, G. West, and C. Ratti, J. R. Soc. Interface **11** (2014).
[3] M. Catanzaro, M. Boguña, and R. Pastor-Satorras, Phys. Rev. E **71**, 027103 (2005).
[4] When referring to Europe in this letter, 31 countries are taken into consideration. The names of the countries are as follows: Belgium, France, Bulgaria, Bosnia Herzegovina, Croatia, Germany, Hungary, Finland, Denmark, Netherlands, Portugal, Latvia, Lithuania, Luxembourg, Romania, Poland, Greece, Estonia, Italy, Albania, Czech Republic, Cyprus, Austria, Ireland, Spain, Macedonia, Slovakia, Malta, Slovenia, United Kingdom, Sweden

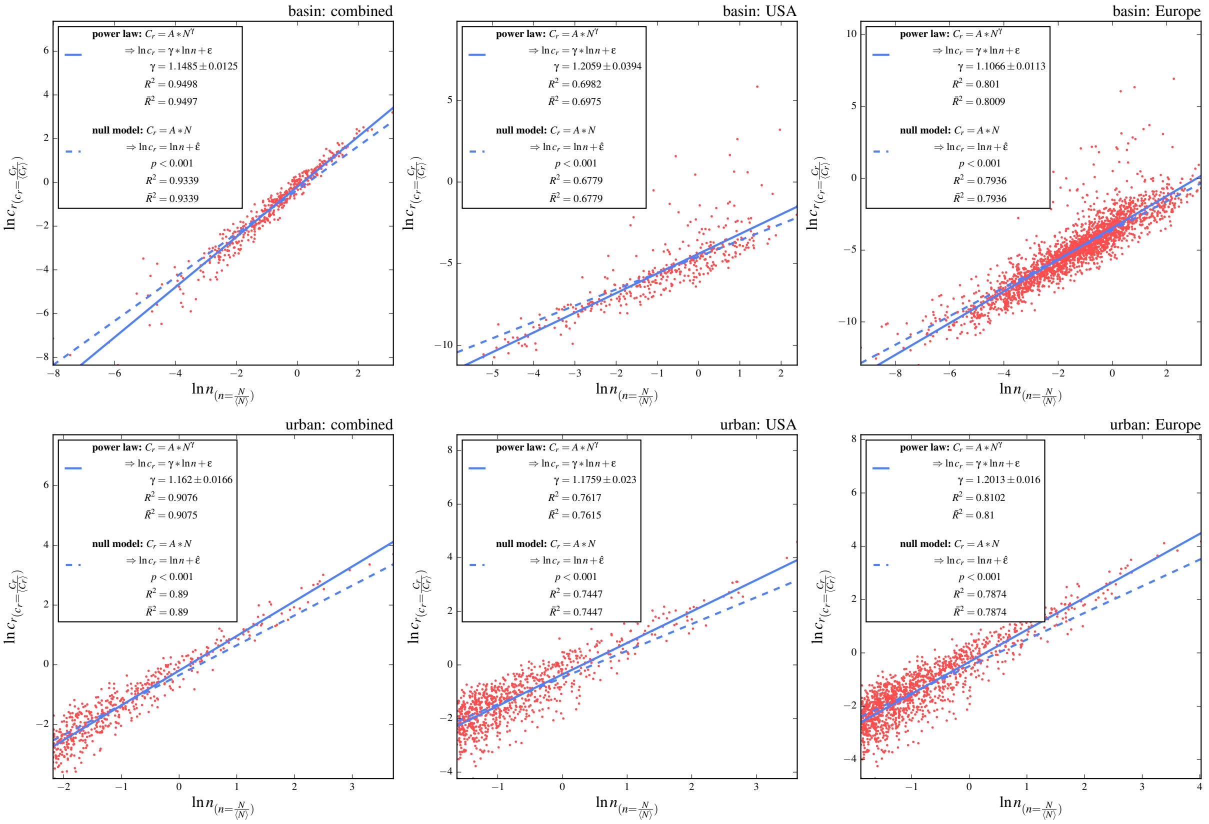

FIG. S1: Top row: rescaled cumulative degree C_r against population N, measured between 13 129 406 Twitter users distributed across 2371 basins in 205 countries (left panel). Center and right panels show the dependency of C_r on N restricted to the Twitter users in the US and Europe. Bottom row: rescaled cumulative degree against population, measured between 4 606 444 Twitter users in 1344 metropolitan areas in 31 countries (left panel). Center and right panels show the dependency of C_r on N restricted to the Twitter users in the US and Europe. We normalized the values of C_r and N by their average to compare the results across different countries. In each panel we show the results of the super-linear and linear model fit, the corresponding $\hat{R}^2$ and the p-value of the Student's t test on the difference between the slopes. In all cases the user-user interactions are bounded within the geographical areas.

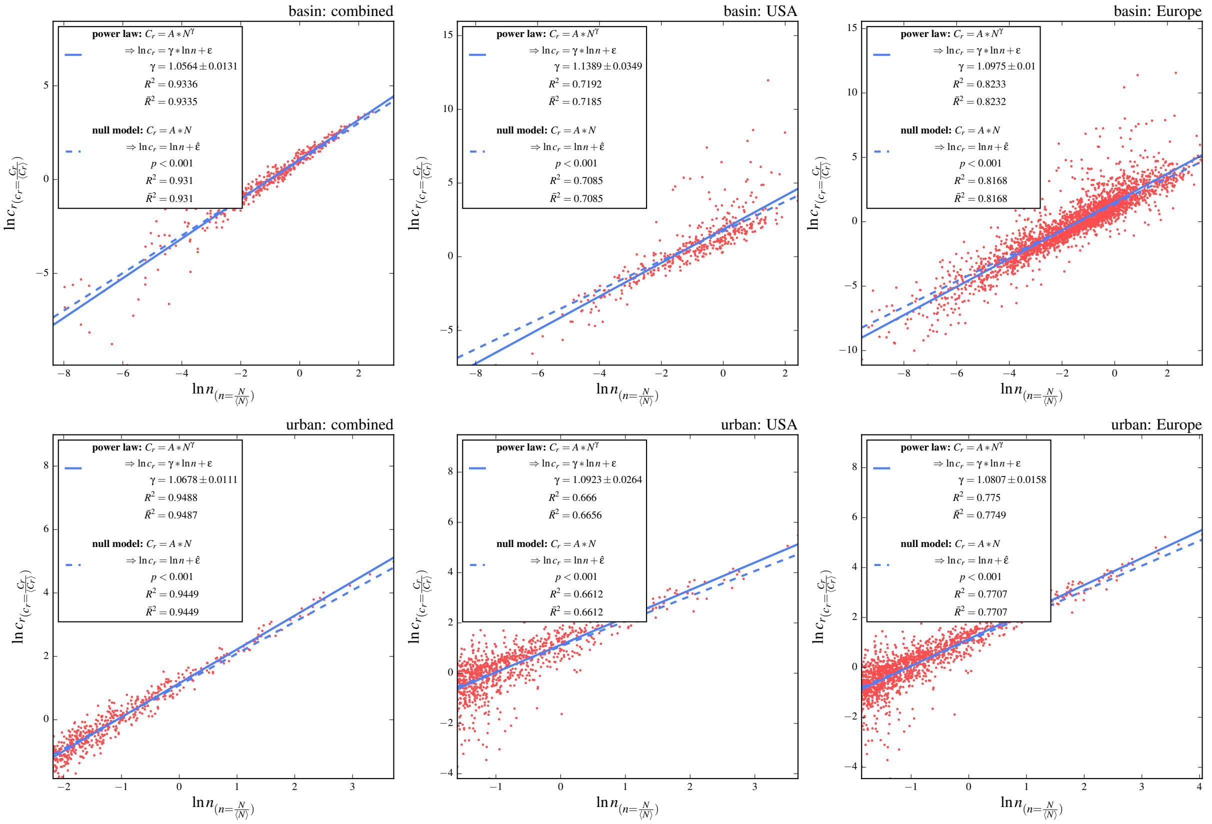

FIG. S2: Top row: rescaled cumulative degree C_r against population N, measured between 13 129 406 Twitter users distributed across 2371 basins in 205 countries (left panel). Center and right panels show the dependency of C_r on N restricted to the Twitter users in the US and Europe. Bottom row: rescaled cumulative degree against population, measured between 4 606 444 Twitter users in 1344 metropolitan areas in 31 countries (left panel). Center and right panels show the dependency of C_r on N restricted to the Twitter users in the US and Europe. We normalized the values of C_r and N by their average to compare the results across different countries. In each panel we show the results of the super-linear and linear model fit, the corresponding $\hat{R}^2$ and the p-value of the Student's t test on the difference between the slopes. In all cases the user-user interactions are not limited within geographical areas.

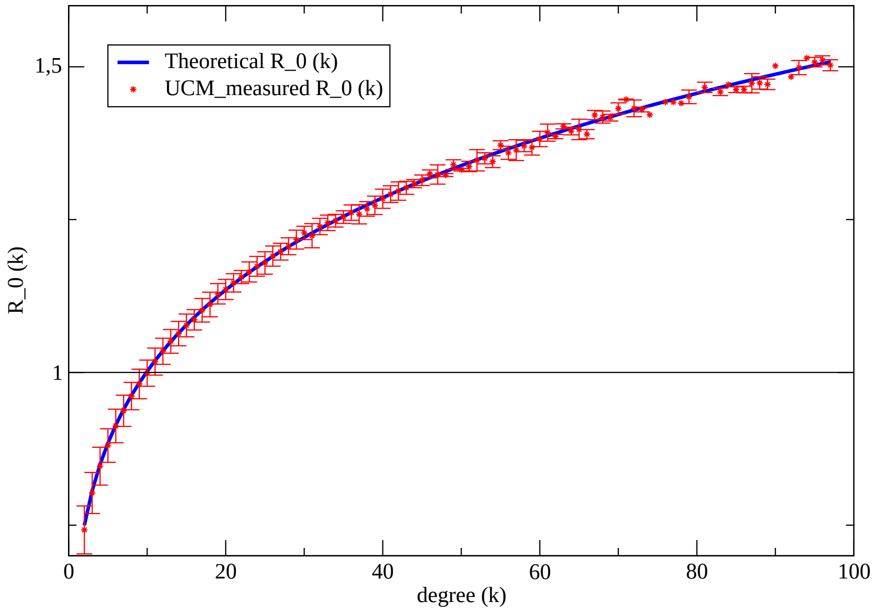

FIG. S3: We show that, for some values of the parameters, $R_0(k)$ can be smaller than 1 for some degree classes and greater than 1 for the other classes. The theoretical curve has been calculated on Eq. 37, while the empiric curve is measured averaging the values of $R_0(k)$ in each degree class in a synthetic network (obtained with the uncorrelated configuration model algorithm [3]) at the stationary state. In this plot we used: $\overline{N} = 10^3,\ \theta = 0.5,\ P(k) \sim k^{-2.1}|_2^{\sqrt{V}}, \eta = 0.12,\ \mu = 0.35,\ V = 10^4$.

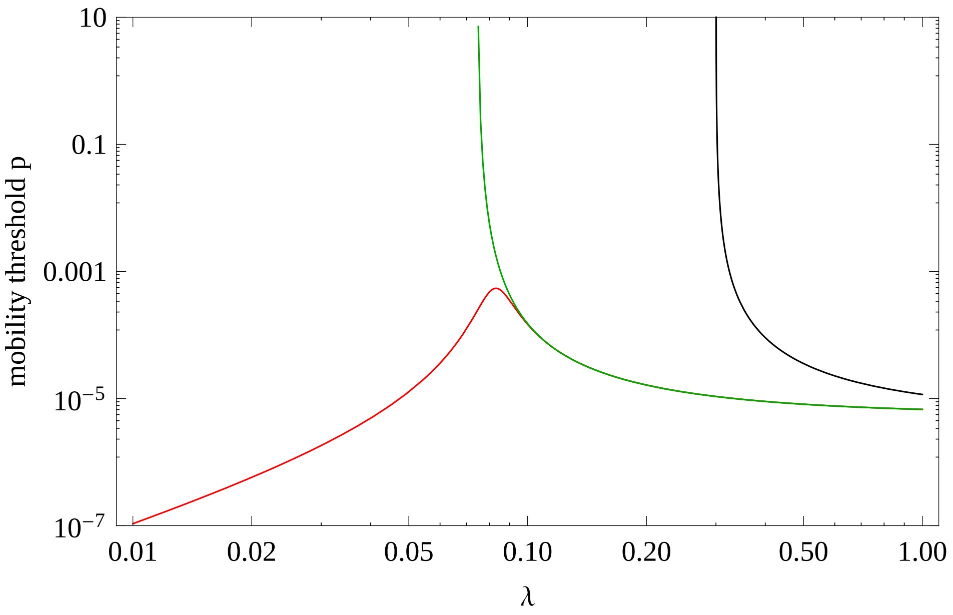

FIG. S4: Comparison between the mobility threshold $p\bar{N}$ with (red) and without (black) scaling of the contacts with population size. In green we plot the new threshold that accounts also for the $R_0(k) < 1$. In the plot we used: $\overline{N} = 10^3,\ \theta = 0.5,\ P(k) \sim k^{-2.1}|_2^{\sqrt{V}} \eta = 0.12,\ \mu = 0.3,\ V = 10^5$.

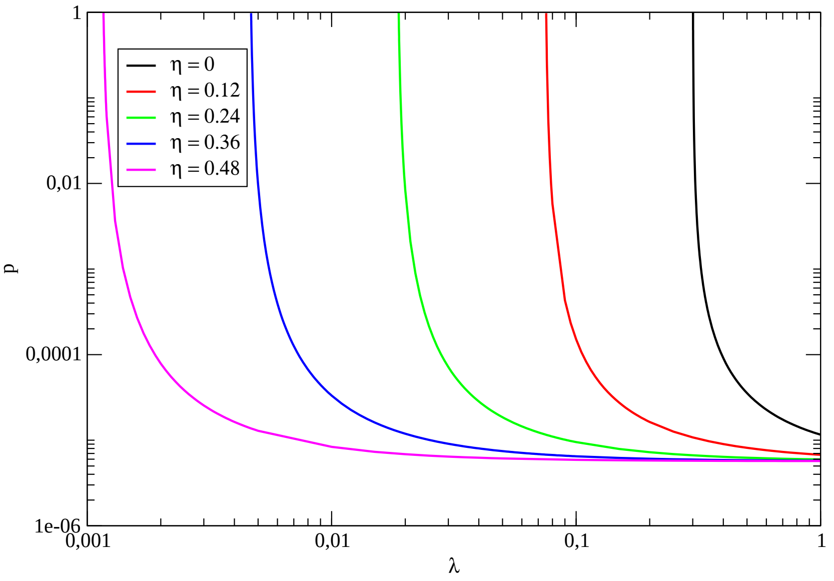

FIG. S5: Phase space $R_*(p, \lambda)$ for different values of η . In the plot we used: $\overline{N} = 10^3,\ \theta = 0.5,\ P(k) \sim k^{-2.1}|_2^{\sqrt{V}},\ \mu = 0.3,\ V = 10^5$.

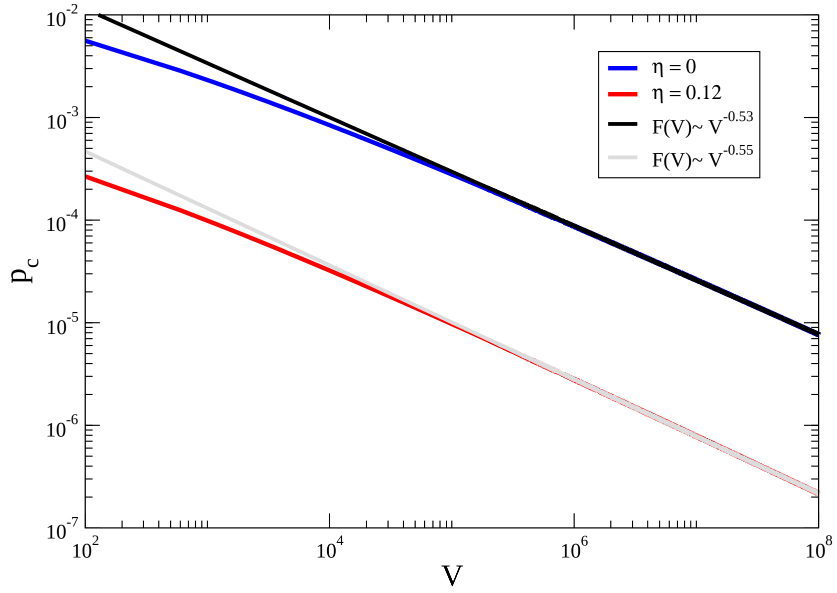

FIG. S6: Scaling behavior of p_c with V for two different values of η. In the plot we used: $\overline{N} = 10^3,\ \theta = 0.5,\ P(k) \sim k^{-2.1}|_2^{\sqrt{V}},\ \mu = 0.3, \lambda = 0.35$. The black and grey lines describe power-law fits. The theoretical prediction for the scaling is $p_c \propto V^{\frac{1-\gamma}{2}} = V^{-0.55}$

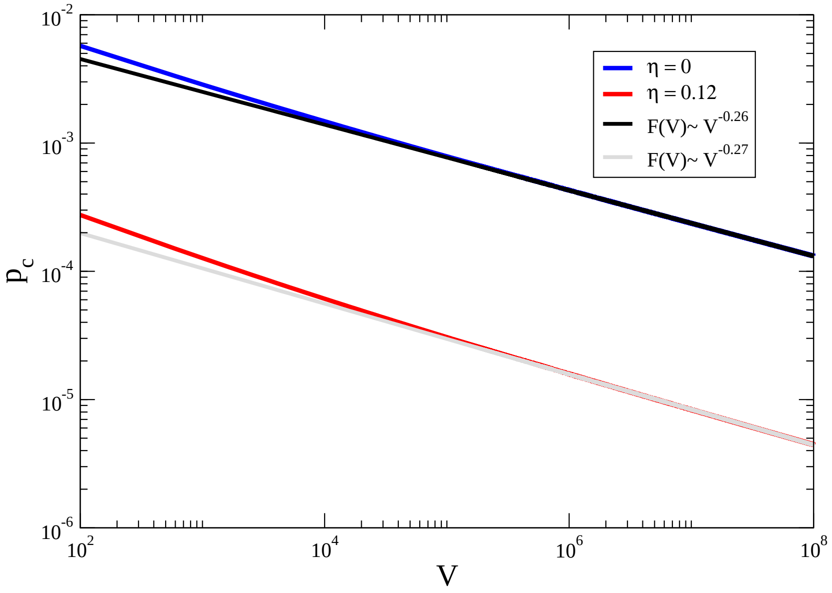

FIG. S7: Scaling behavior of p_c with V for two different values of η. In the plot we used: $\overline{N} = 10^3$, $\theta = 0.5$, $P(k) \sim k^{-1.5}|_2^{\sqrt{V}}$, $\mu = 0.3, \lambda = 0.35$. The black and grey lines describe power-law fits. The theoretical prediction for the scaling is $p_c \propto V^{\frac{1-\gamma}{2}} = V^{-0.25}$

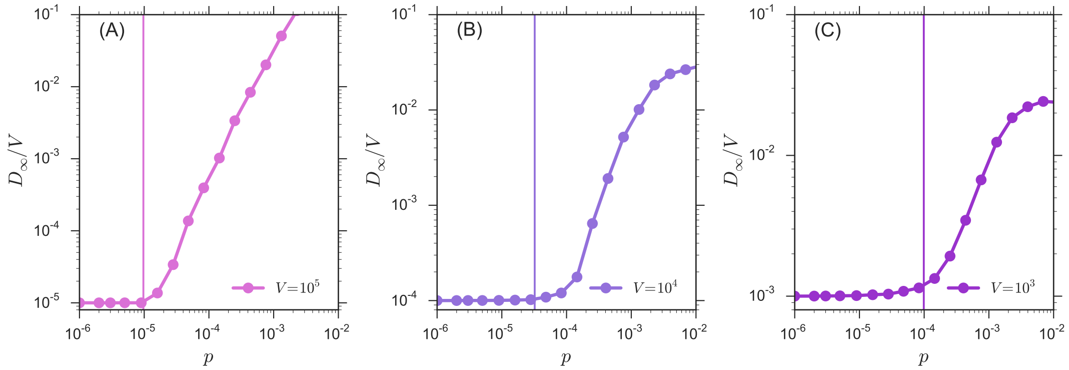

FIG. S8: D_∞/V as a function of p for different values of the network size V. We considered $V = 10^5$ (panel A), $V = 10^4$ (panel B), and $V = 10^3$ (panel C). In the simulations we used: $\overline{N} = 10^3$, $\theta = 0.5$, $P(k) \sim k^{-2.1}|_2^{\sqrt{V}}$, $\mu = 0.3, \lambda = 0.35$. The vertical lines indicate the theoretical prediction for the mobility threshold.
